# Simple technique for evacuation of traumatic subcutaneous haematomas under tension

**DOI:** 10.1186/1471-227X-5-11

**Published:** 2005-12-13

**Authors:** George Chami, Belinda Chami, Edward Hatley, Hossam Dabis

**Affiliations:** 1 Department of Orthopaedics, Epsom General Hospital, Epsom, Surrey, UK

## Abstract

**Background:**

Traumatic subcutaneous haematomas are common cases in the accident and emergency department. Occasionally, urgent evacuation is required to prevent skin necrosis.

**Methods:**

We present a simple and safe technique, based on a principle similar to liposuction to evacuate traumatic subcutaneous haematomas under tension. Instruments readily available in the accident and emergency department are used without the need of general anaesthesia.

**Results:**

The technique was performed in six cases without complication such as infection or re-collection of the haematoma under tension. We present two typical scenarios where urgent evacuation was indicated and the technique performed.

**Conclusion:**

The technique is useful as an urgent and safe procedure in the accident and emergency department setting.

## Background

Traumatic subcutaneous haematomas are common conditions. Patients on anticoagulation treatment are at particular risk. Occasionally, large collections of blood accumulate and the pressure within the haematomas can exceed the blood pressure in the dermal and subdermal capillaries which may result in large areas of necrosis of the overlying skin [1]. In these cases an urgent evacuation of the haematoma is indicated mainly to release the tension over the skin. However, due to clot formation the haematomas are very difficult to evacuate by aspiration so incision and evacuation is usually performed which commonly warrants urgent admission and general anaesthesia.

## Methods

**Figure 1:**
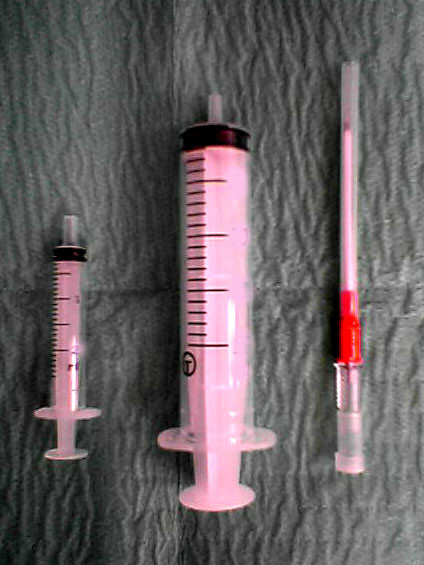
**Instrumentations**. Instruments needed for the procedure.

**Figure 2:**
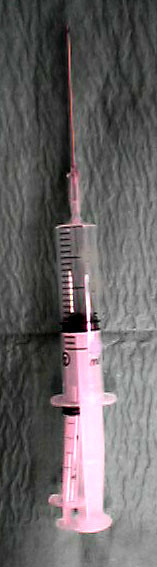
**Assembly of the instruments**. The 50 mls syringe is withdrawn and a smaller syringe is inserted between the withdrawn plunger and the syringe base allowing continued high suction through the cannula without the need of a suction pump.

**Figure 3:**
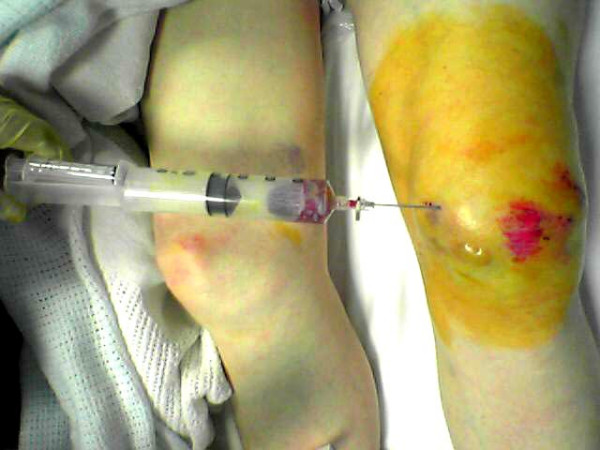
**Case 2**. Large traumatic pre-patella haematomas. The skin was compromised and thus drained using the simple technique as described.

The technique is based on a principle similar to liposuction. It requires three simple instruments: a 50 mls syringe, a 10 mls syringe and a 16 Gauge cannula as shown in figure 1. The 50 mls syringe is attached to a 16 Gauge cannula as shown in figure 2 and inserted into the subcutaneous haematoma as in figure 3. The cannula is advanced in an oblique sharp angle to the skin to prevent continuous leakage from the insertion site after the procedure. The syringe is then withdrawn to a point where a 10 ml syringe can be inserted between the withdrawn plunger and the syringe base as in figure 2 and figure 3. This allows continued high suction through the cannula without the need of a suction pump. The haematoma is then evacuated by a "to and fro" movement of the cannula through the haematoma breaking it up into small pieces which are sucked out into the syringe. The syringe is emptied and the procedure can be repeated until satisfactory release of the skin tension is achieved. A simple dressing covered with a compression bandage is applied. An oral antibiotic is prescribed for three days.

## Results

The technique has been used to evacuate six cases without any complications or evidence of infection. The procedure was sufficient to release the tension of the skin in all cases. We will present two typical examples where this technique was the only available option for urgent release of the skin tension:

### Case 1

A 43 years old lady presented to the accident and emergency department with an elbow injury. On examination there was a large subcutaneous haematoma over the olecranon, the skin vascularity was compromised and urgent release of the skin tension was indicated. A radiograph revealed a displaced olecranon fracture but without any bony fragments stretching the skin. The operating theatres were not available for a further 4–5 hours and the patient had just eaten. Due to the presence of a fracture the procedure of surgical incision to evacuate the haematoma within the accident and emergency department setting could bear an unacceptable high risk of infection. Whilst on the other hand waiting for the operating theatres to become available and allowing enough time from the patients last meal for general anaesthesia may result in a large area of skin necrosis overlying a fracture. By using this technique the haematoma was evacuated within 5–10 minutes by the single insertion of a 16 gauge needle. No analgesia was used as the needle was inserted into the edge of skin that was already stretched. Minimal discomfort was felt during evacuation but dramatic pain relief followed from release of the tension. The patient was subsequently admitted for open reduction and internal fixation of the fracture which was performed after 24 hours in optimal conditions.

### Case 2

A 75 years old gentleman presented to the accident and emergency department eight hours after falling onto his knees. Initially he was able to walk but later his mobility became limited due to knee pain not responding to oral analgesia. His knee examination revealed a large haematoma in the prepatella bursa with the overlying skin vascularity compromised and the skin covered with blisters as shown in figure 3. A radiograph of the knee showed no fractures. The patient was on warfarin due to a previous pulmonary embolism and the lNR level was 3. An urgent evacuation of the haematoma was required to release the tension. However, patient was not starved or prepared for general anaesthesia and the haematoma size was too large for the use of local anaesthesia. Formal incision and drainage with an INR of 3 could trigger a haemorrhage requiring extension of the incision to control the blood loss and accordingly full preparation by blood cross matching and general anaesthesia is then needed. Waiting until the INR is within the operative range will require 24–48 hours and the patient is likely to end up with a large area of skin necrosis. An attempt of simple aspiration of the haematoma by the accident and emergency staff failed. Thus, the technique was used and the tension released within 10 minutes in the accident and emergency department. A compression bandage was applied and the knee was rested for few days in a splint. The case was followed up and there was no evidence of re-collection of the haematoma under tension, skin necrosis or infection. Accordingly formal incision and drainage was not required at a later stage.

## Discussion

Evacuation of the haematoma using a formal liposuction apparatus has been described in lectures for different anatomical positions of haematomas [1-3] and the technique usually requires hospitalisation of the patient. However, the liposuction instruments are not readily available in the accident and emergency department or in hospitals without a plastic surgical department and the surgeons therefore resort to incision and drainage using an open technique.

A Yankauer sucker and suction pump has been described for pre-tibial haematomas in elderly patients [4]. However, the technique needs a surgical incision that is large enough for the suction handle to be introduced deep into the haematoma cavity which has only been tried for pre-tibial haematomas in elderly patients. Such a large incision and introduction of a large instrument into the haematoma cavity could bear an unacceptable risk of infection and is likely to be painful, especially if the haematoma is connected to a fracture.

A simple minimally invasive technique is described in this paper based on a liposuction principle using instruments readily available and can be easily used in accident and emergency, the wards or outpatient departments. It is a procedure allowing urgent evacuation of the haematoma under tension and preventing pressure necrosis of the overlying skin. In appropriate cases hospitalisation is not required and the patient can be treated on an outpatient basis.

Although the haematoma cannot be evacuated completely using this technique we did not find any complications including re-collection of the haematoma under tension even in patients on anticoagulants. This could have been due to two factors: Firstly, the technique is minimally invasive and unlikely to trigger a major re-bleed. Secondly, any minor re-bleed can be controlled by the clotting factors present in the residue clots of the haematoma and the compression bandage. Although we did not see any evidence of infection after the procedure we emphasise that this technique despite its simplicity should be carried out under aseptic conditions. An oral prophylactic antibiotic is also advised.

## Conclusion

We present a simple, fast method to evacuate traumatic subcutaneous haematomas under tension. We found it very useful as an urgent and safe procedure in the accident and emergency setting. The procedure can be considered as the definitive treatment in appropriate cases and patients can be treated in the outpatients departments.

## Competing interests

The author(s) declare that they have no competing interests.

## Authors' contributions

All authors contributed equally to this work. BC participated in its design and coordination and helped to draft the manuscript. All authors read and approved the final manuscript.

## Pre-publication history

The pre-publication history for this paper can be accessed here:


